# Application of qPCR assays based on haloacids transporter gene *dehp2* for discrimination of *Burkholderia* and *Paraburkholderia*

**DOI:** 10.1186/s12866-019-1411-0

**Published:** 2019-02-11

**Authors:** Xianbin Su, Yi Shi, Ruihong Li, Zhao-Ning Lu, Xin Zou, Jiao-Xiang Wu, Ze-Guang Han

**Affiliations:** 1 Key Laboratory of Systems Biomedicine (Ministry of Education), Shanghai Center for Systems Biomedicine, Shanghai Jiao Tong University, Shanghai, China; 2 Shanghai Quality Safety Centre of Agricultural Products, Shanghai, China

**Keywords:** *Burkholderia*, *Paraburkholderia*, Haloacids transporter, Dehp2, qPCR, Pathogenicity

## Abstract

**Background:**

A major facilitator superfamily transporter Dehp2 was recently shown to be playing an important role in transport and biodegradation of haloacids in *Paraburkholderia caribensis* MBA4, and Dehp2 is phylogenetically conserved in *Burkholderia* sensu lato.

**Results:**

We designed both *Burkholderia* sensu stricto-specific and *Paraburkholderia*-specific qPCR assays based on *dehp2* and 16S rRNA, and validated the qPCR assays in 12 bacterial strains. The qPCR assays could detect single species of *Burkholderia* sensu stricto or *Paraburkholderia* with high sensitivity and discriminate them in mixtures with high specificity over a wide dynamic range of relative concentrations. At relatively lower cost compared with sequencing-based approach, the qPCR assays will facilitate discrimination of *Burkholderia* sensu stricto and *Paraburkholderia* in a large number of samples.

**Conclusions:**

For the first time, we report the utilization of a haloacids transporter gene for discriminative purpose in *Burkholderia* sensu lato. This enables not only quick decision on proper handling of putative pathogenic samples in *Burkholderia* sensu stricto group but also future exploitation of relevant species in *Paraburkholderia* group for haloacids biodegradation purposes.

**Electronic supplementary material:**

The online version of this article (10.1186/s12866-019-1411-0) contains supplementary material, which is available to authorized users.

## Background

*Burkholderia* sensu lato includes versatile members that have dramatic different living-styles and occupy diverse ecological niches [1–6]. The most infamous species are *Burkholderia pseudomallei* and *Burkholderia mallei* which cause melioidosis and glanders in animals and humans [7–10]. Besides that, *Burkholderia cepacia* complex (Bcc) includes many closely-related opportunistic pathogens such as *Burkholderia cenocepacia* and *Burkholderia multivorans* [11–14]. There are also many species isolated from the environment with biotechnological application potentials, such as plant growth promotion, antibiotics production, and biodegradation of pollutants [4, 5, 15–17], which are generally defined as “plant-beneficial-environmental (PBE) cluster” [18–20]. Phylogenetic analysis based on single gene such as 16S rRNA, *recA*, *fur*, *acdS*, *hisA* and *rpsU* have revealed the complexity of their evolutionary relationships [21–26]. Based on assessment of conserved sequence indels, a new *Paraburkholderia* genus was created to include the diverse environmental isolates while *Burkholderia* sensu stricto includes *B. mallei*, *B. pseudomallei*, Bcc members and other pathogenic members [27]. The complicated taxonomy of *Burkholderia* sensu lato has attracted great attention, and large scale phylogenomic study has been suggested [28]. In accordance with this suggestion, a recent study systematically analyzed the conserved sequences in 92 *Burkholderia* sensu lato species and demonstrated the existence of 5 lineages: *Burkholderia* sensu stricto, *Paraburkholderia*, *Caballeronia*, the newly described genus *Robbsia* [29], and the lineage represented by *Paraburkholderia rhizoxinica* [30]. Assessment of the phylogenetic position of new isolates or samples containing *Burkholderia* or *Paraburkholderia* could be useful for further analysis, and there have been such reports based on molecular techniques such as PCR [22, 31–34], multi-locus sequence typing (MLST) [35–38] and qPCR assays [39–41]. As qPCR could sensitively quantitate the target and is accessible to more places compared with sequencing-based approaches, it has great application potentials in the phylogenetic studies of *Burkholderia* sensu lato.

*Paraburkholderia caribensis* (formerly *Burkholderia caribensis*) MBA4 is a bacterium with the ability to degrade environmental pollutant haloacids [42]. Besides the hydrolytic enzyme dehalogenase, membrane transporters that mediate active uptake of haloacids are also important for effective biodegradation [43–45]. Although structurally similar, haloacetate is transported with a different system compared with acetate [46]. We have recently revealed that *P. caribensis* MBA4 harbors two haloacids transporters, Deh4p and Dehp2, which show overlapping but not identical substrate specificities [45, 47]. The expression of *dehp2* is strictly regulated in response to the presence of haloacids in its growing environment, and the ~ 100 bp upstream non-coding region of *dehp2* is highly conserved in *Burkholderia* sensu lato [48]. Three strains from other environmental *Paraburkholderia* species, namely *P. caribensis* LMG 18531, *Paraburkholderia phymatum* (formerly *Burkholderia phymatum*) STM815, and *Paraburkholderia xenovorans* (formerly *Burkholderia xenovorans*) LB400, gained the ability to degrade haloacids with the introduction of dehalogenase Deh4a, and haloacids-inducible haloacids transport activities were observed in accordance with haloacids-inducible expressions of *dehp2* orthologs, strongly suggesting their roles as haloacids transporters [16]. Dehp2 thus represents a group of conserved transporters in *Burkholderia* sensu lato, and the phylogenetic tree based on Dehp2 clearly show two clades corresponding well to *Burkholderia* sensu stricto and *Paraburkholderia* [43]. This provides the ground of exploiting Dehp2 for discrimination of *Burkholderia* sensu stricto and *Paraburkholderia*.

In this study, we tested the utilization of *dehp2* as a phylogenetic marker for quick discrimination of putative pathogenic/opportunistic pathogenic *Burkholderia* sensu stricto and mainly environmental-derived *Paraburkholderia*. We designed qPCR assays that target the regions of *dehp2* and 16S rRNA conserved in *Burkholderia* sensu stricto or *Paraburkholderia* and validated their performance in 12 strains of bacteria from *Burkholderia* sensu lato. The results showed that *dehp2* could be used as a discriminative marker similarly as 16S rRNA, and assays based on both markers produced more reliable results with high specificity and sensitivity. This is the first report on the utilization of a haloacids transporter as a discriminative marker in *Burkholderia* sensu lato, which will be useful for further clinical or biotechnological studies.

## Results

### Quick detection of *Burkholderia* sensu stricto and *Paraburkholderia* by qPCR assays

As described previously, both 16S rRNA and *dehp2* phylogenetic trees display two major groups which could discriminate *Burkholderia* sensu stricto and *Paraburkholderia* species [5, 43]. We first designed three pairs of qPCR primers based on 16S rRNA: 16S-F1/R1 to target the region conserved in *Burkholderia* sensu lato, 16S-F2/R2 to target the region conserved in *Burkholderia* sensu stricto, and 16S-F3/R3 to target the region conserved in *Paraburkholderia*. Similarly, we designed dehp2-F6/R6 and dehp2-F7/R7 to target *Burkholderia* sensu stricto-specific and *Paraburkholderia*-specific regions of *dehp2*. To ensure that the primers cover all the sequence variations, we incorporated degenerate bases during primer design, which have been a common practice in microbial studies [49], such as microbial population taxonomy [50], diversity of functional genes related to antibiotic or arsenite resistance, etc. [51, 52].

**Fig. 1:**
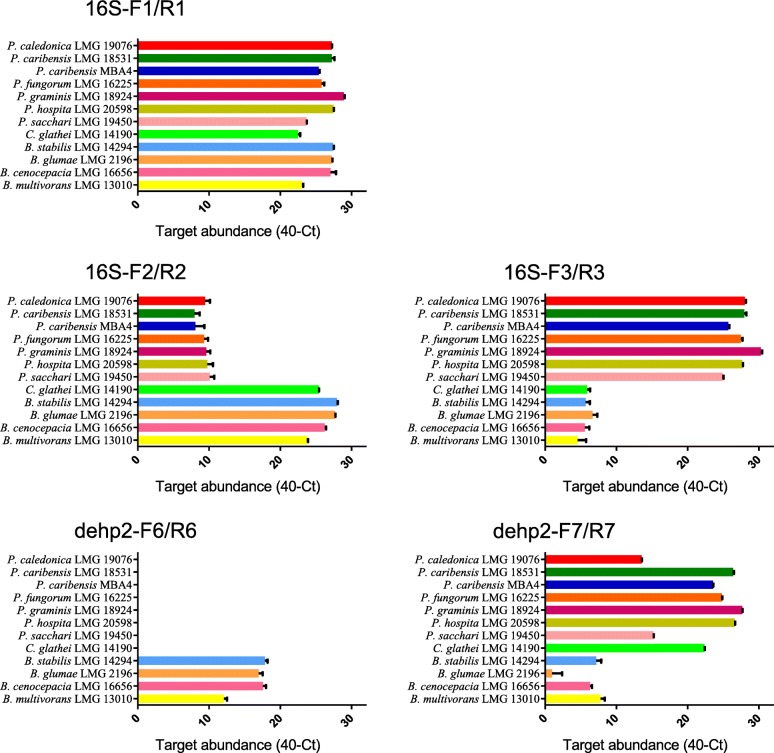
qPCR assays to discriminate *Burkholderia* and *Paraburkholderia* species bacteria. qPCR analysis was carried out for the 12 strains with the 5 pairs of primers: 16S-F1/R1, 16S-F2/R2, 16S-F3/R3, dehp2-F6/R6 and dehp2-F7/R7. Target abundance was measured by 40 minus Ct value for each condition. Results shown are the means of three replicates with the error bars representing the standard deviations

We first analyzed the performances of the 5 pairs of primers against the 12 strains using a single strain as the template for each qPCR reaction (Fig. 1). For the *Burkholderia* sensu lato-conserved primer pair 16S-F1/R1, all 12 strains showed similar amplification efficiency. The *Burkholderia* sensu stricto-specific primer pair 16S-F2/R2 had statistically higher amplification efficiency with the 4 *Burkholderia* strains and *C. glathei* LMG 14190, while the *Paraburkholderia*-specific 16S-F3/R3 primer pair showed statistically higher amplification efficiency with the 7 *Paraburkholderia* strains. For *dehp2*, the *Burkholderia* sensu stricto-specific primer pair dehp2-F6/R6 had statistically higher amplification efficiency with the 4 *Burkholderia* strains, while the *Paraburkholderia*-specific dehp2-F7/R7 primer pair showed statistically higher amplification efficiency with the 7 *Paraburkholderia* strains and *C*. *glathei* LMG 14190. It should be pointed out that the amplification efficiency of qPCR assays will be affected by degenerate primers, but the amplification efficiencies of our discriminative qPCR assays show such dramatic differences between *Burkholderia* sensu stricto and *Paraburkholderia* group that they still enable effective discrimination of the two group even with the use of degenerate primers. The performances of primers targeting *dehp2* were comparable to 16S rRNA-based primers, suggesting *dehp2* could be used as a marker for discrimination of *Burkholderia* sensu stricto and *Paraburkholderia*.

The above results clearly showed that the 4 pairs of discriminative primers work well in regard to their ability to discriminate *Burkholderia* sensu stricto and *Paraburkholderia*. The interesting species is *C. glathei*, as qPCR patterns based on 16S rRNA assays were similar to *Burkholderia*, while qPCR patterns based on *dehp2* assays were similar to *Paraburkholderia*. This seemingly contradictory results showed the uniqueness of this species, which is supported by its recent transfer to a new genus *Caballeronia* [53].

### Specific discrimination of *Burkholderia* and *Paraburkholderia* from mixtures by qPCR assays

**Fig. 2:**
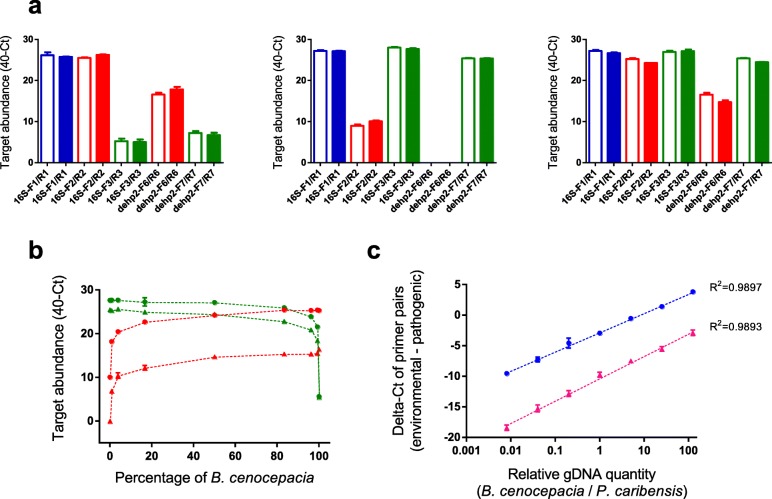
qPCR assays of mixtures of *Burkholderia* and *Paraburkholderia* species bacteria. **a** qPCR assays with the 5 pairs of primers were validated against 1:1 mixture of *B. cenocepacia* LMG 16656 and *B. multivorans* LMG 13010 (left panel), 1:1 mixture of *P. caledonica* LMG 19076 and *P. caribensis* LMG 18531 (middle panel), and 1:1 mixture of *B. cenocepacia* LMG 16656 and *P. caribensis* LMG 18531 (right panel). The empty bars showed the theoretic values computed from qPCR data of single species from Fig. 1, while filled bars showed real detected values. **b** qPCR assays with the 4 pairs of primers (16S-F2/R2,; 16S-F3/R3,; dehp2-F6/R6,; dehp2-F7/R7,) were validated against different concentration combinations of *B. cenocepacia* LMG 16656 and *P. caribensis* LMG 18531. **c** The relationship of Delta-Ct values of *Burkholderia* sensu stricto-specific or *Paraburkholderia*-specific primer pairs using 16S rRNA () or *dehp2* () with the relative proportion of *Burkholderia* and *Paraburkholderia* targets. Results shown are the means of three replicates with the error bars representing the standard deviations

The above results showed that the qPCR assays are able to discriminate whether the samples contain *Burkholderia* or *Paraburkholderia* species when we used a single bacterial species as the qPCR template. For environmental or clinical samples collected without further cultivation and isolation, it is common that they contain different bacterial species. To test whether our qPCR assays are capable of specific discrimination of *Burkholderia* or *Paraburkholderia* species, we mimicked such conditions by preparing mixtures of gDNA from known species. We first tested three conditions: equal concentration mixture of 2 *Burkholderia* species, mixture of 2 *Paraburkholderia* species, and mixture of 1 *Burkholderia* species and 1 *Paraburkholderia* species. Our qPCR assays could clearly tell whether there are only *Burkholderia*, only *Paraburkholderia*, or both genera in the samples, which are also consistent with values calculated from qPCR results of single species (Fig. 2a).

In addition to the equal concentration mixtures, we further assessed mixtures of *Burkholderia* and *Paraburkholderia* species at constant total concentration but different ratios. qPCR showed that for relative ratios of 1:125, 1:25, 1:5, 1:1, 5:1, 25:1 and 125:1 of *B. cenocepacia* LMG 16656 to *P. caribensis* LMG 18531, all the 4 pairs of discriminative primers exhibited specific detection (Fig. 2b). We then used the Delta-Ct values between the *Burkholderia* sensu stricto-specific and *Paraburkholderia*-specific primers (16S-F3/R3 vs. 16S-F2/R2, dehp2-F7/R7 vs. dehp2-F6/R6) to monitor their ability to discriminate the two genera. This approach could also avoid possible interference by other closely related bacteria. For the dynamic range tested, high correlation coefficients were observed for Delta-Ct values against different ratios of the two genera for both 16S rRNA and *dehp2* (Fig. 2c). The results showed that our qPCR assays can specifically discriminate *Burkholderia* and *Paraburkholderia* species with a wide dynamic range of relative ratios.

### Illustrative visualization of the qPCR data by hierarchical clustering (HC) and principal component analysis (PCA)

**Fig. 3:**
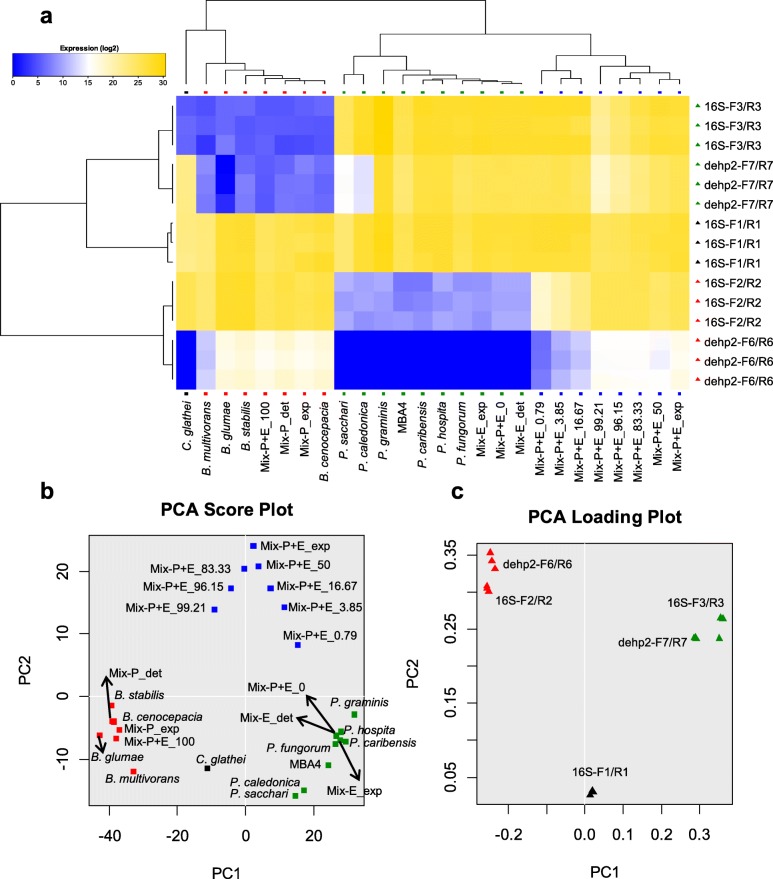
Hierarchical clustering (HC) and principal component analysis (PCA) of the qPCR assays. The data used in the Figs. 1 and 2 were combined and further analyzed with HC and PCA. **a** Heat-map based on HC. The samples and genes were differently colored based on the genus they belong. PCA score plot (**b**) and PCA loading plot (**c**) of the results are shown. For sample groups: *Burkholderia*,; *Paraburkholderia*,; *Burkholderia* + *Paraburkholderia*,; other, . For gene groups: *Burkholderia* sensu stricto-specific,; *Paraburkholderia*-specific,; *Burkholderia* sensu lato-conserved, . For sample names, ‘Mix-P’, ‘Mix-E’ and ‘Mix-E + P’ indicate mixture of two *Burkholderia* species, two *Paraburkholderia* species, and one *Burkholderia* and one *Paraburkholderia* species, respectively; ‘_exp’ and ‘_det’ indicate expected and detected values; the numbers after ‘Mix-E + P’ indicate the percentage of *B. cenocepacia* LMG 16656 in the mixtures. All three replicates of qPCR are shown

We then used HC and PCA to analyze the qPCR results for more straightforward visualization. For the qPCR data collected against single species or mixtures as described above, we first used HC to construct a heat-map. Both the primer pairs and samples were clustered relevant to the genus they belong, facilitating easy interpretation of the results (Fig. 3a). The uniqueness of *C. glathei* LMG 14190 is also shown in the heat-map. For PCA score plot, the positions of the mixtures are straightforward display of their relative proportion of *Burkholderia* or *Paraburkholderia* (Fig. 3b). For PCA loading plot, the positions of the primer pairs also indicate whether they are targeting *Burkholderia* sensu lato-conserved, *Burkholderia* sensu stricto-specific or *Paraburkholderia*-specific region (Fig. 3c). In general, HC and PCA displays of the qPCR results facilitate easy detection of the presence and relative quantity of *Burkholderia* and *Paraburkholderia* species.

### High sensitivity of the qPCR assays for detection of *Burkholderia* and *Paraburkholderia*

**Fig. 4:**
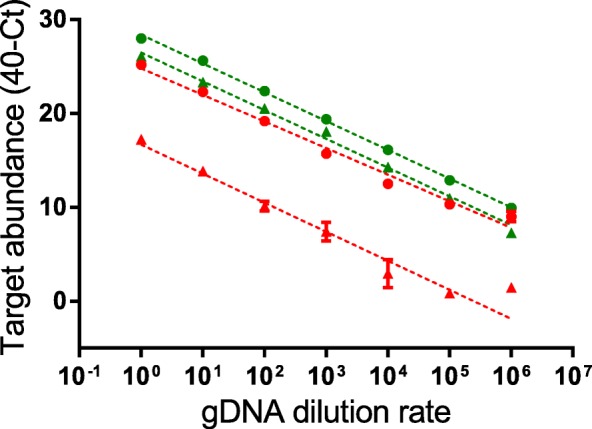
Detection sensitivity of the qPCR assays. The sensitivities of the 4 pairs of primers (16S-F2/R2,; 16S-F3/R3,; dehp2-F6/R6,; dehp2-F7/R7,) were checked against serial diluted bacterial gDNA (~ 50 ng μl^− 1^ for both *B. cenocepacia* LMG 16656 and *P. caribensis* LMG 18531) at the following rate: 10^1^, 10^2^, 10^3^, 10^4^, 10^5^ and 10^6^. Results shown are the means of three replicates with the error bars representing the standard deviations

Our qPCR assays are able to specifically discriminate *Burkholderia* and *Paraburkholderia* species in complicated samples, and the amplification specificities of the 4 discriminative PCR assays were further confirmed by using gDNA of *E. coli* DH5α as negative control (Additional file 1: Figure S1). We then checked their sensitivity at detecting the target species. We prepared serial diluted bacterial gDNA of *Burkholderia* and *Paraburkholderia*, and for a dynamic range of six orders of magnitude, all 4 pairs of primers worked consistently (Fig. 4). The targets were approaching several-copies to even single-copy per qPCR assay for the lowest concentration tested (calculated to be < 10 copies μl^− 1^), and all primer pairs were performing well except for *Burkholderia* sensu stricto-specific dehp2-F7/R7, which is relatively less efficient compared with the others. This is reasonable, as haloacids transporter gene is a good marker for environmental rather than pathogenic species. The above results showed that our qPCR assays are highly sensitive for detection of low-quantity of *Burkholderia* and *Paraburkholderia* target in the samples.

## Discussion

Considering the pathogenic potentials of some species in *Burkholderia* sensu lato, it is useful to first have a quick assessment before further analysis such as sequencing, especially when there is a large number of specimens. As some bacteria may not be cultured successfully, qPCR assay should be more sensitive than culture-dependent detection methods. On the other hand, although high throughput sequencing is more powerful to give a global view of genomes, qPCR can actually show very consistent results towards specific targets at much lower cost [54]. Moreover, qPCR is also easier to handle and accessible to more places compared with sequencing-based identification approaches. With these advantages, there have been many qPCR based methods for detection or discrimination purposes in *Burkholderia* sensu lato. For example, qPCR assay based on Type III Secretion System enabled quick and accurate identification of *B. pseudomallei* [39], and qPCR assay Bu550 that targets a 7 kb locus was able to discriminate *B. ubonensis* from its close neighbor *B. pseudomallei* [55]. Multi-target qPCR assays were able to detect the presence of Bcc members at the resolution of species from sputum specimens [40]. Our study represents the first application of a haloacids transporter gene to discriminate *Burkholderia* and *Paraburkholderia*, which will be useful for not only detecting pathogenic species but also screening environmental species that can be exploited for bioremediation of haloacids.

It has been suggested that phylogenetic relationship could not be reliably established based on single gene [56], and the inclusion of more independent targets increased the sensitivity compared with single target method for identification of *B. pseudomallei* [41, 57]. In this study, we also considered this issue and utilized both the haloacids transporter gene *dehp2* and 16S rRNA. Indeed, there were in-consistence between results obtained from assays based on *dehp2* and 16S rRNA in regard to *C. glathei*, which was transferred to a novel genus recently [53], further supporting the reliability of the assays. Moreover, our qPCR assays were designed to target regions conserved in both pathogenic *Burkholderia* sensu stricto species and environmental *Paraburkholderia* species, and the Delta-Ct values between *Burkholderia* sensu stricto-specific and *Paraburkholderia*-specific assays could be utilized to calculate the relative ratio of the two genera in mixtures. The combination of qPCR assays that target both *Burkholderia*-specific and *Paraburkholderia*-specific regions in *dehp2* and 16S rRNA could rule out possible inference by other related environmental bacteria. High sensitivity and specificity of such assays were observed for both *dehp2* and 16S rRNA over a broad dynamic range of mixtures from both genera.

Our previous work has established Dehp2 as a haloacids transporter [43, 47], and the successful application of this gene to discriminate *Burkholderia* sensu stricto and *Paraburkholderia* further proved its importance for *Burkholderia* sensu lato. As haloacids are not the natural nutrients for pathogenic *Burkholderia* species and even some of the environmental *Paraburkholderia* species, the presence of this transporter showed gene expression rewiring and adaption potentials of bacteria to their living environment. Further analysis of this transporter gene among the two genera, such as evolutionary analysis of key amino acid residues and comparative assessment of the promoter regions between pathogenic and environmental species should provide precious clues for understanding of the transport mechanisms and technological exploitation of relevant species to efficiently degrade haloacids without causing pathogenic risks.

It should be pointed out that, however, qPCR assay is just a first step for full understanding of the bacteria or samples. As has been cautioned, phylogenetic positions not necessarily confirm whether a *Burkholderia* or *Paraburkholderia* species is pathogenic or not [5, 6, 58]. The results based on this qPCR assay should raise our attention to putative pathogenic species that must be handled carefully, and the samples suggested to be in the environmental group should also be systematically assessed before wide technological applications. Another fact that should be emphasized is that we only tested the qPCR assays in 12 bacterial strains, which represent a relatively small sampling of the > 100 of strains from *Burkholderia* sensu lato. Further assessment of the assays in a larger sample pool of *Burkholderia* sensu lato members will be beneficial for the research field and broad applications of the assays.

## Conclusions

In this study we designed qPCR assays based on haloacids transporter Dehp2 as well as 16S rRNA, which enable quick discrimination of *Burkholderia* species and *Paraburkholderia* species with high sensitivity and specificity. Results obtained with the qPCR assays will facilitate more specific handling in regard to the putative pathogenicity of the samples and also exploitation of relevant species for haloacids bioremediation.

## Methods

### Bacterial strains and extraction of genomic DNA

We used 12 strains from 11 species from *Burkholderia* sensu lato in this study, namely *Paraburkholderia caledonica* LMG 19076, *P. caribensis* LMG 18531, *P. caribensis* MBA4, *Paraburkholderia fungorum* LMG 16225, *Paraburkholderia graminis* LMG 18924, *Paraburkholderia hospita* LMG 20598, *Paraburkholderia sacchari* LMG 19450, *Caballeronia glathei* LMG 14190, *Burkholderia stabilis* LMG 14294, *Burkholderia glumae* LMG 2196, *B. cenocepacia* LMG 16656 and *B. multivorans* LMG 13010, which were gifts from Molecular Microbiology Laboratory of The University of Hong Kong (Table 1). Genomic DNAs (gDNAs) from the 12 strains were extracted with a G-spin™ Genomic DNA Extraction Kit (iNtRON). The concentrations of the gDNA were adjusted to 50 ~ 100 ng μl^− 1^.

**Table 1:** Bacterial strains used in this study

Bacterial strains	Description	References
*E. coli* DH5α	Negative control for primer validation	Takara
*P. caledonica* LMG 19076	Bacterium isolated from the rhizosphere	[61]
*P. caribensis* LMG 18531	Exopolysaccharide-producing bacterium isolated from vertisol	[62, 63]
*P. caribensis* MBA4	Haloacids-degrading bacterium isolated from soil	[42, 64, 65]
*P. fungorum* LMG 16225	Bacterium isolated from the white-rot fungus	[61]
*P. graminis* LMG 18924	Bacterium isolated from rhizosphere of grasses	[66]
*P. hospita* LMG 20598	Bacterium isolated from B-horizon soil	[67]
*P. sacchari* LMG 19450	Polyhydroxyalkanoate-accumulating bacterium isolated from soil	[68]
*C. glathei* LMG 14190	Bacterium isolated from lateritic soil	[53, 66, 69]
*B. stabilis* LMG 14294	Pathogenic bacterium isolated from sputum of a cystic fibrosis patient, Bcc member	[70]
*B. glumae* LMG 2196	Plant pathogen	[71]
*B. cenocepacia* LMG 16656	Pathogenic bacterium isolated from a cystic fibrosis patient, Bcc member	[72]
*B. multivorans* LMG 13010	Pathogenic bacterium isolated from sputum of a cystic fibrosis patient, Bcc member	[73]

### Primer design of 16S rRNA and *dehp2*

The 16S rRNA and *dehp2* sequences from the above described strains were retrieved from NCBI. For the haloacids transporter gene *dehp2*, the sequences are available in 6 species (*P. caribensis*, *P. fungorum*, *B. stabilis*, *B. glumae*, *B. cenocepacia*, and *B. multivorans*), and the sequences are currently unknown in the other 5 species (*P. caledonica*, *P. graminis*, *P. hospita*, *P. sacchari* and *C. glathei*). The sequences of 16S rRNA or *dehp2* were aligned using ClustalW [59]. Three pairs of qPCR primers were then designed based on 16S rRNA: 16S-F1/R1 to target the region conserved in *Burkholderia* sensu lato, 16S-F2/R2 to target the region conserved in *Burkholderia* sensu stricto, and 16S-F3/R3 to target the region conserved in *Paraburkholderia*. We designed dehp2-F6/R6 and dehp2-F7/R7 to target *Burkholderia* sensu stricto-specific and *Paraburkholderia*-specific regions of *dehp2*. The sequences of the 5 pairs of primers are shown in Table 2.

**Table 2:** Primers used in this study

Primers	Sequence (5′ to 3′)^a^
16S-F1	GGTAATACGTAGGGTGC**R**AGCGTT
16S-R1	CAC**M**AATGCAGTTCCCAGGTT**R**AG
16S-F2	GGAGGAATACCGATGGCGAAGG
16S-R2	TTACTAAGGAAATGAATCCCCAACAAC
16S-F3	ACAAGCGGTGGATGATGTGGAT
16S-R3	TGTGTTA**Y**GGCTCCCTTTCGG
dehp2-F6	**R**CA**Y**TCGCCGATGACG**RS**
dehp2-R6	GGA**R**AAGAAGCTCTTGCTGAT**R**T
dehp2-F7	**R**C**M**TGGGGCTGGCGCATT
dehp2-R7	GTCCGG**R**TT**S**GCGATCACGAC

^a^Degenerated bases are shown in bold

### qPCR

qPCR was carried out using SYBR® Premix Ex Taq™ (Clontech) on the StepOnePlus system (Applied Biosystems) with a two-step method: initial denaturation of 95 °C for 30 s; 40 cycles of 95 °C for 5 s and 66 °C for 30 s. A melting curve program was also included to verify the specificity of the amplified products. Three replicates were set up for each condition, and negative controls were also included to monitor possible contaminations. To measure the amplification efficiency, 40 minus Ct values were used, which represent logarithmic transformed target abundance as previous described [54, 60]. To check the ability of combination of primer pairs to discriminate *Burkholderia* sensu stricto and *Paraburkholderia*, the difference between Ct values derived from 16S rRNA primers (Ct_16S-F3/R3_ - Ct_16S-F2/R2_), or between those derived from *dehp2* primers (Ct_dehp2-F7/R7_ - Ct_dehp2-F6/R6_) were calculated. For qPCR reactions, besides the single strains analyzed, we also tested mixtures of different strains. To check the sensitivity of qPCR assays, serial diluted gDNA of *B. cenocepacia* LMG 16656 was used as template for 16S-F2/R2 and dehp2-F6/R6, while serial diluted gDNA of *P. caribensis* LMG 18531 was used as template for 16S-F3/R3 and dehp2-F7/R7.

### Visualization of data by HC and PCA

HC and PCA analysis of the qPCR data were performed using the SINGuLAR™ Analysis Toolset R package (Fluidigm).

## Additional file

Additional file 1: **Figure S1** Validation of the amplification specificity of the discriminative PCR primers. (PDF 226 kb)

## Abbreviations

Bcc: *Burkholderia cepacia* complex
HC: Hierarchical clustering
PCA: Principal component analysis
