# Supplementary material for: Application of qPCR assays based on haloacids transporter gene dehp2 for discrimination of Burkholderia and Paraburkholderia
Source: BMC Microbiol. 2019 Feb 11;19:36. doi: 10.1186/s12866-019-1411-0 (PMC6371555; doi:10.1186/s12866-019-1411-0)
Supplement: Supplementary file 1 — Figure S1 Validation of the amplification specificity of the discriminative PCR primers. (PDF 226 kb) [file 12866_2019_1411_MOESM1_ESM.pdf]

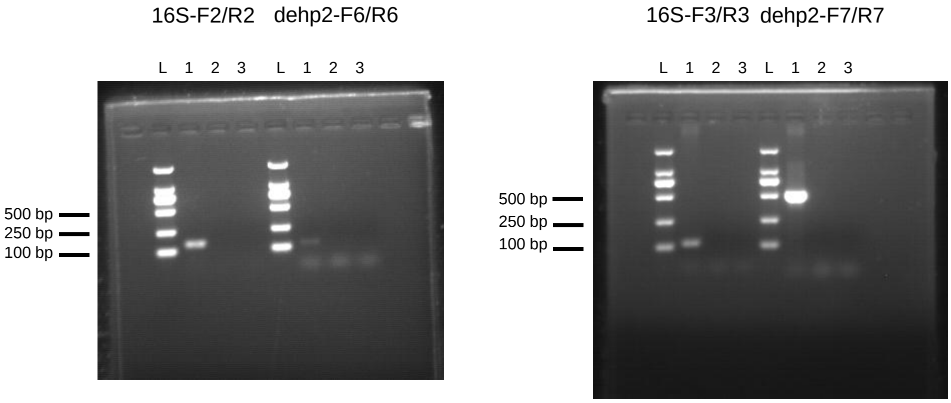

Figure S1 Validation of the amplification specificity of the discriminative PCR primers. For each pair of primers, three PCR reactions are shown. For 16S-F2/R2 and dehp2-F6/R6, ′1′ indicates reaction with gDNA of *B. cenocepacia* LMG 16656 as template; and for 16S-F3/R3 and dehp2-F7/R7, ′1′ indicates reaction with gDNA of *P. caribensis* LMG 18531 as template. ′2′ indicates reaction with gDNA of *E. coli* DH5α as template, ′3′ indicates no template control, and ′L′ indicates the DNA ladder with the sizes of three bands shown on the left.
